# Supplementary material for: Improvement of the Diagnostic Performance of Facial Neuritis Using Contrast-Enhanced 3D T1 Black-Blood Imaging: Comparison with Contrast-Enhanced 3D T1-Spoiled Gradient-Echo Imaging
Source: J Clin Med. 2021 Apr 24;10(9):1850. doi: 10.3390/jcm10091850 (PMC8141108; doi:10.3390/jcm10091850)
Supplement: Supplementary file 1 [file jcm-10-01850-s001.zip › jcm-1178458-supplementary.pdf]

Supplementary Table 1. MR imaging parameters for CE-GRE and T1 BB-FSE sequences

MR Sequence	Parameters	
	Skyra	Verio
CE-GRE		
Repetition time (ms)	16	28
Echo time (ms)	9	5
Flip angle (degree)	25。	18。
Matrix	320 x 320	320 x 272
Field of view (mm)	230 x 230	230 x 230
Section thickness (mm)	0.8	0.8
NEX	1	1
Scan time (minute)	5:01	5:15
T1 BB-FSE		
Repetition time (ms)	700	770
Echo time (ms)	4	21
Flip angle (degree)	variable	variable
Matrix	320 x 272	256 x 212
Field of view (mm)	230 x 230	230 x 230
Section thickness (mm)	0.8	0.8
NEX	2	2
Scan time (minute)	4:51	4:40

*Abbreviations: CE-GRE, contrast enhanced 3D-T1-spoiled gradient echo;

T1 BB-FSE, contrast enhanced 3D T1 black-blood fast spin-echo;

NEX, number of excitations.
